# Harmonizing emotions in the workplace: exploring the interaction between emotional intelligence, positive psychological capital, and flourishing

**DOI:** 10.3389/fpsyg.2023.1343043

**Published:** 2024-01-11

**Authors:** Rosa Isabel Rodrigues, Ana Junça Silva

**Affiliations:** ^1^Instituto Superior de Gestão - Business & Economics School, Lisbon, Portugal; ^2^ISCTE - University Institute of Lisbon, Lisbon, Portugal

**Keywords:** emotional intelligence, positive psychological capital (PsyCap), flourishing at work, emotions at work, positive psychology

## Introduction

The globalized environment has undeniably provided new opportunities for organizational growth. This expansion has brought about various changes that are reflected in the way cultural diversity is managed (Inegbedion et al., 2020). The ability to understand and manage emotions, both one's own and those of others, is essential for building effective relationships in multicultural teams (Brett et al., 2020). Therefore, the importance attributed to emotional intelligence (EI) has come to be considered the key to effective performance, as it contributes to promoting a positive work environment that is reflected in organizational performance (Blázquez Puerta et al., 2021).

According to Kurdi and Hamdy (2020), this type of intelligence plays a particularly crucial role in an individual's adaptation to different spheres of life. Manifesting in situations involving the ability to perceive, express, understand, regulate, and utilize emotions to promote wellbeing, physical and mental health, and overall satisfaction (Brunetto et al., 2023). As a result, organizations are realizing that the technical skills of their employees are no longer sufficient for gaining a competitive advantage (Asbari et al., 2020). They highlight the need for employees to understand their own emotions and those of others to guide their thoughts, behaviors, and actions positively (Diener et al., 2020). Moroń and Biolik-Moroń (2021) add that individuals with high levels of EI are more perseverant and adopt strategies that enable them to face situations with greater confidence in the face of adversity.

## Emotional intelligence and positive psychology: strengthening psychological capital for personal and professional success

EI enables individuals to (i) recognize their own emotions, (ii) manage them efficiently, (iii) self-motivate for the establishment and achievement of goals (personal and professional), (iv) recognize the emotions of others, and (v) manage relationships with others to ensure personal and professional high-quality relationships (Goleman, 1995; O'Connor et al., 2019). Thus, EI is based on the learning of skills such as self-awareness, maintaining optimism, perseverance, empathy, cooperation, and motivation (Junça-Silva et al., 2023). The ability to use emotions to positively direct behavior facilitates adaptation to the environment, overcoming obstacles, reducing conflict scenarios, which is why it is fundamental to achieving success on a personal and professional level (Alonazi, 2020). Consequently, Aderibigbe et al. (2020) note that EI is positively correlated with positive psychological capital (PsyCap). This state of positive individual development characterizes people who: (i) have confidence (self-efficacy) to take and make the effort needed to succeed in their tasks (Kumar et al., 2022); (ii) make positive attributions (optimism) about current and future success (Darvishmotevali and Ali, 2020); (iii) are perseverant in relation to their goals and redirect them if necessary (hope; Ngo, 2021); and (iv) have the ability to overcome obstacles or resist pressure in adverse situations (resilience; Rabenu and Tziner, 2020).

Self-efficacy concerns an individual's conviction regarding their own ability to mobilize motivation, cognitive resources, and necessary courses of action for the successful execution of a specific task in a given context (Sarwar et al., 2017). Optimism, on the other hand, can be defined as the attributional style in which positive events are attributed to permanent and universal internal (personal) causes, while negative events are interpreted based on external, temporary, and specific factors (Clarence et al., 2018). Resilience refers to an individual's capacity to regain balance after a situation of great stress or adversity, resulting from the balance between the tension of risk factors and the ability to confront them (protective factors). It is the ability to recover from conflicting, adverse, or failed situations while maintaining balance and responsibility (Pathak and Joshi, 2020). Finally, hope characterizes the process that reflects the belief that a goal is achievable. It is the belief that one can set goals, find a way to achieve them, and motivate oneself to reach them (Fang et al., 2020).

In this context, Luthans and Broad (2020) argue that positive psychological capabilities are an essential tool for promoting wellbeing, serving as a type of capital that allows individuals to navigate obstacles in a balanced way, whether at the individual, group, or organizational level. Following the same line, Zahra and Kee (2019) state that individuals with adequate levels of PsyCap have more resources to overcome difficulties, face challenges, and achieve success. In light of this, Moroń and Biolik-Moroń (2021) suggest that self-efficacy, optimism, resilience, and hope increase the ability to understand and regulate emotional experiences and can be considered personal protective resources in the face of adversity. The conservation of resources theory (Hobfoll, 1989) explains the relationship between PsyCap and EI. According to Hobfoll et al. (2018), resources are invaluable to individuals, helping them face the adversities, demands, and uncertainties of daily life. These resources prevent the emergence of negative emotions or negative affective states, such as stress or anxiety (Junça-Silva et al., 2023). Thus, PsyCap, as a resource, can not only help individuals identify and manage their emotions but also contribute to establishing strong and lasting relationships (Costa et al., 2021).

The dimensions of PsyCap are renewable and operate synergistically, so individuals demonstrating relevant values in them are more flexible (Pathak and Joshi, 2020), adaptable, and self-aware (Costa et al., 2021). The rapid adaptation to changes in their contextual environment, facilitated by the levels of self-regulation stemming from Emotional Intelligence (EI), leads individuals to regulate their own behavior to reduce the discrepancy between their expectations and the situation they find themselves in (Goldsby et al., 2021). For instance, in a sample of individuals who had recently lost their jobs, Chen and Lim (2012) demonstrated a positive association between PsyCap and adaptive coping strategies focused on problem-solving and active job search. Other studies have suggested that PsyCap, as a positive personal resource, enables individuals to be happier and achieve higher levels of flourishing (Santisi et al., 2020).

## Psychological synergies

Flourishing is an indicator of psychological and subjective wellbeing (Diener et al., 2010). Wellbeing refers to individuals' subjective assessments of the quality of their lives based on their personal experiences, relationships, feelings, and overall functioning (Brazeau et al., 2020). However, a conceptual distinction has been made in the literature between subjective (or hedonic) wellbeing and psychological (or eudaimonic) wellbeing. Subjective wellbeing includes a person's emotional responses, satisfaction with specific domains, and overall judgment of life satisfaction (Huta, 2020). In contrast, psychological wellbeing is defined in terms of psychological functioning and personal growth, including how individuals interact with their environment (Ryff, 1989; Ryff et al., 2020). More recently, Seligman (2011) emphasized the need to integrate both approaches, considering that both denote different but important aspects of wellbeing. The author combined various components of both aspects and proposed a theory of wellbeing focused on flourishing. This concept was introduced to describe the desirable state in which both hedonic and eudaimonic components are simultaneously present (VanderWeele, 2020). According to Diener et al. (2010) and Bakracheva (2020), an individual is said to be in a state of flourishing when they can satisfy their universal psychological needs, find meaning and purpose in life, engage in relevant activities, maintain optimism, establish and maintain positive social relationships, and have self-esteem and feelings of competence.

## Discussion

The relationship between PsyCap and EI may stem from the positive psychological states achieved through flourishing. According to Freire et al. (2020), PsyCap is a long-term predictor of flourishing and mental health. Chevalier et al. (2021) also showed that PsyCap plays a relevant role in how individuals can self-manage emotionally. This self-management largely depends on self-esteem, generalized feelings of competence, and the meaning attributed to various dimensions of life. Similarly, the ability to remain optimistic and engage in important and pleasurable activities avoids the accumulation of stress and contributes to improving each individual's emotional management capacity (Darvishmotevali and Ali, 2020). PsyCap allows for the development of resources that enhance an individual's emotional capacity (Imran and Shahnawaz, 2020). Thereby, minimizing the negative impact of adversities and daily demands on wellbeing and enabling the maximization of psychological flourishing states (Rabenu and Tziner, 2020).

The dynamic interaction between Psycap, EI and flourishing shapes human behavior. The ability to recognize and regulate emotions, a characteristic of emotional intelligence, acts as a catalyst for strengthening the Psycap, promoting resilience, optimism, and self-efficacy (Widowati and Satrya, 2023). These elements, in turn, contribute to a positive state of mind, which is essential for flourishing. This synergistic process maximizes individuals' potential, enabling them to attain overall wellbeing and fulfillment across diverse aspects of their lives, such as social and professional domains (Abdolmohammadi et al., 2021).

## Author contributions

RR: Supervision, Visualization, Writing—original draft, Writing—review & editing. AJ: Supervision, Visualization, Writing—original draft, Writing—review & editing.
